# Effects of Childhood Nutrition Education from School and Family on Eating Habits of Japanese Adults

**DOI:** 10.3390/nu14122517

**Published:** 2022-06-17

**Authors:** Mizuki Kuwahara, Wonsub Eum

**Affiliations:** Department of English, The University of Kitakyushu, Kitakyushu 802-8577, Japan; a9111049@foreign.kitakyu-u.ac.jp

**Keywords:** nutrition education, eating habits, family intervention, Japan

## Abstract

Since the Basic Law of Shokuiku (nutrition education) was established in 2005, the Japanese government has been promoting nutrition education among children to encourage better eating habits. This study analyzes the 2019 survey data on people’s attitudes towards nutrition education, in order to elucidate the relationship between the results of nutrition education, attitude towards nutrition education and proper eating habits, and the experience of nutrition education. The results reveal that people who received nutrition education at elementary school and middle school tend to have a more positive attitude towards nutrition education. In addition, family conversation on foods during elementary school years has a positive effect on nutritionally balanced eating behavior.

## 1. Introduction

This study investigates the relationship between eating habits and nutrition education by looking at the cross-sectional survey data among Japanese citizens. In recent years, despite rising life expectancy in Japan, there are still lots of health issues closely related to unhealthy eating behaviors [1]. According to *The National Health and Nutrition Survey in Japan* in 2020 by the Japanese Ministry of Health, Labor and Welfare, the percentage of obese males was 31.8%, and that of underweight women aged 20 to 29 years was 20.7%. These health issues can lead to serious side effects, including heart disease and diabetes in cases of obesity, and irregular periods and osteoporosis in cases of underweight, eventually resulting in high societal costs [2].

As a response, since the *Basic Law on Shokuiku* (nutrition education) was enacted in 2005, the Japanese Ministry of Health, Labor and Welfare has promoted healthy eating behavior by defining the basic idea of nutrition education and showing the role of each stakeholder. Among the seven key fundamental concepts stated in the law, two concepts aim to promote nutrition education at home and nutrition education at schools. The Japanese government also initiated a two-stage national health promotion regime called Health Japan 21 in 2000 and 2012, which further promoted nutrition education for children by national and local governments, schools and individuals [3]. This policy assures the health of future generations by increasing the number of children who carefully eat three meals a day, or increasing the frequency of family eating. As such, the Japanese government has promoted nutrition education to nurture people who practice healthy eating by acquiring food-related knowledge and skills, and to educate children about improving their physical and mental health [4].

A number of previous empirical studies on nutrition education have commonly pointed out that health and nutrition education in school improves children’s health knowledge and nutrient intakes and promotes higher physical activity, thus leading to healthier conditions of children, such as reduced childhood obesity [5,6]. Studies from Japanese data also report similar results from different types of nutrition education programs, such as Japanese Food Guide *Spinning Top* [7,8]. This guides how much of each food type, such as grains and vegetables, each person should consume each day, for nutrition education for elementary school and high school students. Their results show that the students who received nutrition education using this method had higher understanding of and improvements in dietary behavior.

Still, families, teachers, and society should be involved in nutrition education together. Children from infancy to adolescents are surrounded by various people who take important roles for their growth and nutritional intakes [9,10]. Yet, nutrition education is usually led by the diet and nutrition educators in schools, even though they collaborate with other stakeholders by sharing knowledge about food and eating habits to parents, or creating local field experience opportunities of food production and cooking. However, the roles of families in nutrition education should not be overlooked, as children’s behaviors are closely related to the hours they spend with their parents [11]. Specifically, positive eating behaviors last longer with parent nutrition education [12], and parents are both a source of authority and role models [13]. Therefore, it is necessary to specifically analyze each stakeholder’s role in school-based nutrition education.

Theoretically, parent nutrition education is beneficial, as children need support from their family members in order to make behavior changes, including changes in their eating habits [12]. A number of empirical studies provided evidence of a positive relationship between eating with families and desirable healthy dietary intake [14,15]. These studies argue that if children eat with their families, they will eat more fruits and vegetables. Similar evidence is also found with Japanese cases, as children eating with family members are more likely to eat more nutritionally balanced diets than those who eat alone [1,16,17], and are likely to have higher recognition of healthy eating [18]. However, these studies focus on the effects of nutrition education on children, rather than the longer-term effects of nutrition education during childhood on adults, and some studies provide mixed results on the relationship [19].

In short, previous studies found that there is a clear and positive relationship between nutrition education, from both school and family, and the eating behavior of nutrition education recipients. However, there are a number of research gaps in the field of nutrition education and its social effects. First, most studies focus on the behavioral changes in students from nutrition education, rather than its effects on adult eating behaviors. Second, there is a need to clarify the effects of nutrition education by the education type and the age of students during the education. To fill these gaps, this study distinguishes two types of nutrition education from school and family, and provides empirical evidence on the effects of nutrition education on adults, represented by their attitude towards nutrition education and how often they have balanced meals. The analysis further divides the nutrition education by the ages of the recipients at the time of education, in order to provide policy implications to nutrition education at specific ages. 

The rest of the study is aligned as follows: Section 2 describes the data and the methodology to approach the research questions on the relationship between nutrition education and healthy eating habits. Section 3 presents the results of the analysis, and Section 4 discusses the implications of the results and the limitations of this study. Lastly, Section 5 ends the study with a conclusion.

## 2. Materials and Methods

In order to analyze the effectiveness of nutrition education on adults’ attitudes to nutrition education and eating habits, this study uses the Japanese survey data on nutrition education. Since 2005, the Cabinet office of the Japanese government has conducted the preliminary Special Public Opinion Survey on nutrition education, and since 2016, the Ministry of Agriculture, Forestry and Fisheries has been responsible for this survey. Database of this survey is available upon personal request from the Center for Social Research and Data Archives, Institute of Social Science, the University of Tokyo. Among the surveys on nutrition education across different years, this study uses the most up-to-date cross-sectional data of the year 2019, with 3000 participants aged over 20 and 1721 respondents. This survey uses the sample selected by stratified, random, two-stage sampling to represent the Japanese population. The sample of 3000 participants is adjusted by the size of the population in each region, and the number of survey sites are proportionally distributed across the regions to represent the population. The questionnaire includes questions not only on eating behavior and nutrition education, but also on other food-related topics, such as food loss and food safety. It also includes demographic information such as age, gender, marriage status, number of household members, occupations, and regions. The data are not longitudinal and there are limitations in analyzing the causality, but the data provide credible answers for quantitative analysis as professional staffs conduct face-to-face interviews with the respondents [20].

The survey includes 26 types of questions on the attitudes towards food and nutrition, and eating habits. For example, these questions ask whether the respondents have interests in nutrition education, their current eating behaviors, their eating behaviors during childhood, and their attitude towards the prevention of lifestyle disease and the safety of food. In addition, this survey provides data on the experience of nutrition education in elementary school, middle school, and high school. The nutrition education includes in-class education such as experience of agriculture and farming field works and lectures from instructors, and at-home education such as conversations with family members about food and family’s effort to provide healthy foods. Both in-class and at-home nutrition educations bring change in eating behaviors of children, but as they have distinct challenges in practice [12,21], this study aims to provide specific implications for practice by distinguishing the effects of the two types of education.

To measure the interests in diets, this study uses two specific questions as dependent variables: interests in nutrition education and mindset towards healthy eating habits. The independent variables are divided into the nutrition education from school (participation in farming experience and instructions by teachers) and that from home (conversations with family about food while eating and family’s effort to cook likable food). Considering the fact that education at different ages can bring different results [22], this study includes the responses on the nutrition education from school and home at different ages, grouped by the respondents’ experience in elementary schools, junior high schools, and high schools.

The sample used in this study only includes the 1535 participants who answered the questions without answering as they do not know. As eating behavior and attitude towards nutrition education can be influenced by a number of socio-demographic factors, it is essential to control for the confounders. Logistic regressions are widely used among public health studies, as they can control for multiple potential confounders with a large sample size [23]. Following previous studies on nutrition education and eating behaviors [24,25], a logistic regression model was adopted to predict the effects of nutrition education on the binomial dependent variable of attitude towards nutrition education. In addition, an ordered logistic regression model [20] was adopted to predict the effects of nutrition education on the ordinal dependent variable of attitude towards balanced eating behaviors. The issue of multicollinearity among the explanatory variables was modest (vif = 2.76). All statistical analyses were conducted using STATA version 14.0.

## 3. Results

### 3.1. Sample Characteristics

**Figure 1:**
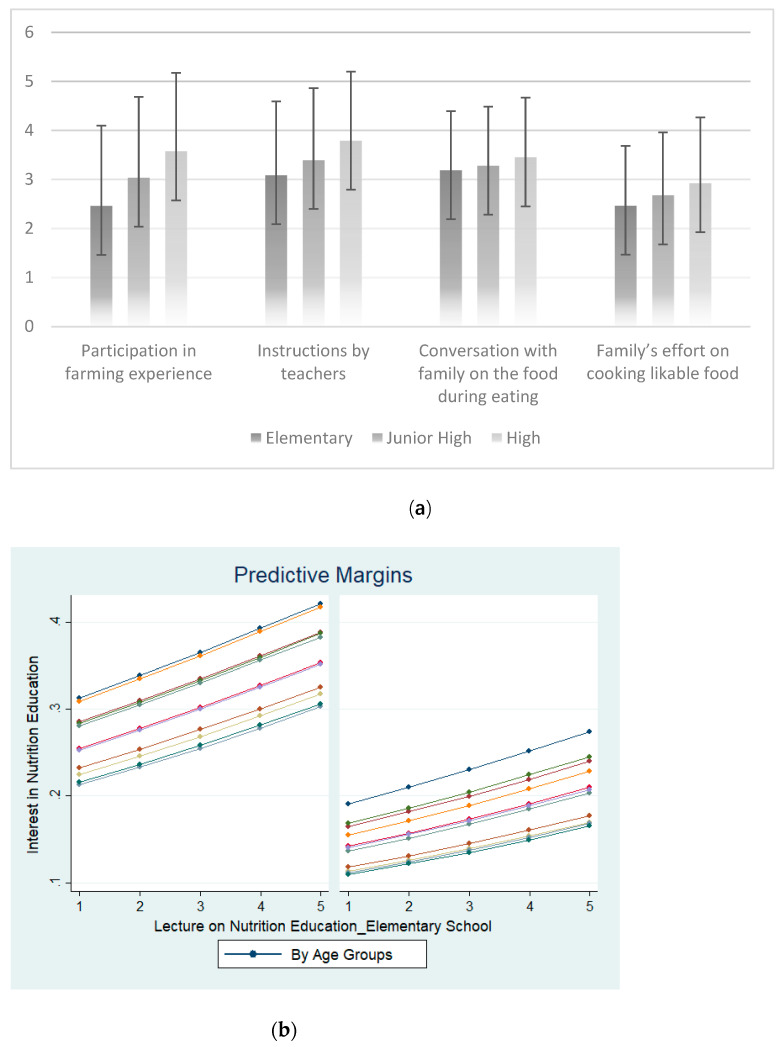
(**a**) Effect of nutrition education instruction by instructors at elementary school on interest in nutrition education, by gender and by age groups. (**b**) Effect of nutrition education instruction by instructors at elementary school on interest in nutrition education, by gender and by age groups. Left shows the results of male respondents and right shows the results of female respondents. The 1–5 scales reflect the degree of the respondents’ experiences. 1 = strongly agree, 2 = agree a little, 3 = neither agree nor disagree, 4 = disagree a little, and 5 = strongly disagree.

This section presents the statistical and econometric results on the analysis of the relationship between nutrition education and the aftermath of nutrition education, represented by the interest in nutrition education and frequency of having nutritionally balanced meals. Table 1 and Table 2 show the summary of the sample in this study, and Figure 1a illustrates the summary statistics of the key independent variables related to nutrition education.

### 3.2. Nutrition Education Factors Influencing Eating Habits

Table 3 presents the results of the logistic regression on the effects of nutrition education during school years on the attitude towards nutrition education. It is noticeable that experience of nutrition education, in the forms of instructions by teachers in classrooms during elementary school and middle school, has a positive influence on the attitude towards nutrition education, whereas the instructions by teachers in high school years did not show a significant effect. This shows that nutrition education for younger students can leave a positive image of nutrition education in the future. On the other hand, the nutrition education from family, such as family conversation about foods and cooking efforts of the family, did not have a significant effect on the attitude towards nutrition education. Regarding gender and age, female and higher-aged groups showed more positive attitudes towards nutrition education.

**Figure 2:**
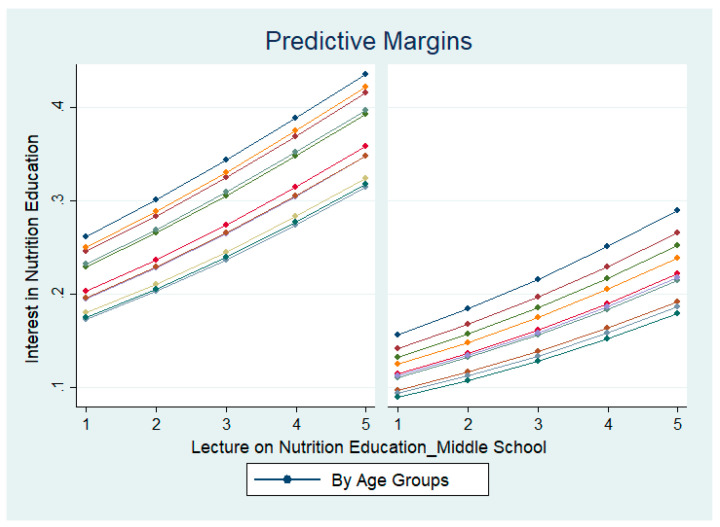
Effect of nutrition education instruction by instructors at middle school on interest in nutrition education, by gender and by age groups. Left shows the results of male respondents and right shows the results of female respondents. The 1–5 scales reflect the degree of the respondents’ experiences. 1 = strongly agree, 2 = agree a little, 3 = neither agree nor disagree, 4 = disagree a little, and 5 = strongly disagree.

As both gender and age had strong effects on the attitude towards nutrition education, the next step further clarifies whether instructions of teachers lead to a positive attitude towards nutrition education across different subgroups of gender and age groups. Figure 1b and Figure 2 show the predictive margins on the effects of instructions during elementary school and middle school, respectively, on the attitude towards nutrition education. Although there are differences in the degree of effects, both figures show similar upward-sloping patterns across both gender and diverse age groups. These results indicate that there is a positive relationship between a positive attitude towards nutrition education and the experience of nutrition education instructions at elementary and middle school.

Table 4 shows the results of ordered logistic regression on the effects of nutrition education during school years on having nutritionally balanced meals. The difference between this analysis and the previous analysis on the attitude towards nutrition education is that the nutrition education from schools, such as farming experience or instructions by teachers, did not have a significant influence on the frequency of having nutritionally balanced meals. Rather, the conversations with families during elementary school years showed a positive and significant impact on balanced eating habits in the future. It is also noteworthy that both gender and age had significant influences on nutritionally balanced eating habits; again, female and higher-aged groups were more likely to have nutritionally balanced meals frequently. These results may come from the socio-demographic differences in occupations, lifestyles, and social status of the respondents at different ages and for different genders.

**Figure 3:**
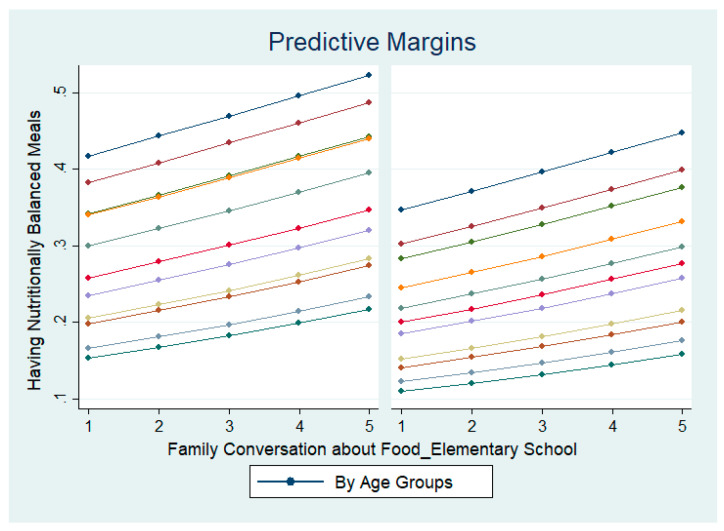
Effect of family conversation about food during elementary school years on having nutritionally balanced meals, by gender and by age groups. Left shows the results of male respondents and right shows the results of female respondents. The 1–5 scales reflect the degree of the respondents’ experiences. 1 = strongly agree, 2 = agree a little, 3 = neither agree nor disagree, 4 = disagree a little, and 5 = strongly disagree.

As was the case for the effect of nutrition education instructions on positive attitudes towards nutrition education, it is necessary to investigate whether family conversations during elementary school years lead to having nutritionally balanced meals, across different gender and age groups. Figure 3 presents the results of predictive margins across gender and age groups. Although there are differences in degree across gender and age groups, upward-sloping patterns can be observed in general. These results show that there is a positive relationship between the experience of family conversations about food during elementary school years and the frequency of having nutritionally balanced meals in the future, emphasizing the importance of family-led nutrition education to foster healthy eating habits.

## 4. Discussion

The results show that family eating at elementary school age affects eating habits at older ages, while food and nutrition education at school does not. One of the possible reasons for this is that such education programs at school are one-time events for students. On the other hand, family eating is a daily routine, and children are more likely to be engaged in the topics of health and nutrition with family members. Hence, the experience of family eating, and even what is talked about on the topics of food, bring similar educational effects to nutrition education. From the analysis, this study reveals that active food-related interactions with family members can bring the results of what nutrition education programs aim to achieve. 

However, the current nutrition education policies in Japan consider parents as the supportive actors of education. According to the White Paper of Nutrition Education, the Japanese government mainly tries to increase the number of diet and nutrition teachers in school. Surely, one of the roles of these teachers is receiving interest about nutrition education from parents, and letting them participate in children’s nutrition education at home. The Japanese government is also trying to promote nutrition education at home by providing various information to parents. However, the current approaches have certain drawbacks. First, there is limited evidence of a positive relationship between parental knowledge about nutrition and healthy nutritional intakes [26]. In addition, if parents do not actively try to gather information on nutrition education by themselves, the effect of nutrition education is limited, as the information is less accessible and the responsibility of nutrition education solely depends on the parents’ attention. Under the current policies, nutrition education at home is a voluntary step by family members.

Therefore, future nutrition education policies in Japan should incorporate further participation from family members. For example, it is important to secure a sufficient number of nutrition teachers so that they can keep track of the long-term family eating behaviors of children. By doing so, nutrition education can be more effective in emphasizing the importance of family eating and healthy eating to not only students, but also their family members. Moreover, instead of only focusing on the nutrition education from school and the instructors, providing more opportunities for family events related to food production, cooking, or eating would promote participation of family members in nutrition education [20]. The successful implementation of such policies would require close cooperation among various interested parties, such as local farmers, local governments, and family members. Furthermore, policies encouraging nutrition education from families should also target double-income households, as children’s lifestyles heavily depend on parental working hours [11], and encourage engagement in frequent food-related conversations and activities. 

There are three limitations in this study. First, this paper does not consider the regional differences in nutrition education and students’ attitudes towards it. For example, students who live in agricultural areas may have more opportunities to participate in nutrition education, such as food production, compared to other students who do not live in such areas. In addition, some students whose family members are involved in agriculture may have a more positive attitude towards nutrition education. Second, nutrition education changes over time (e.g., frequency of field experience, number of students per instructor, and lecture materials), so the aftermath of nutrition education can be different depending on which education the students received [27]. As this study uses a dataset ranging from the age of 20s to over 70s, the effects of different nutrition education are not clearly addressed. Considering these limitations, future research with consideration of regional and time differences in nutrition education and recipients would provide further policy implications to design tailored nutrition education programs [28] for students with specific backgrounds. Third, as the *Basic Law of Shokuiku* was implemented in 2005, there are respondents that did not receive nutrition education after the enactment of the law. Therefore, the quality or the level of nutrition education experienced by the respondents may differ by age groups. Additionally, as found in the analysis result, age is a significant factor that affects both eating behavior and attitude towards nutrition education. Further studies to assess the impact of the nutrition education policy would need to differentiate the types and levels of nutrition education from school, and the effects of education on distinct age groups.

## 5. Conclusions

Despite various studies being conducted on nutrition education, most studies focused on the effect of education on the students’ behaviors, or the education conducted in schools. In order to fill this research gap, this study analyzes the effect of two types of nutrition education from school and family on adult behaviors, based on the cross-sectional Japanese survey data on the dietary behaviors from 2019. The results lead to two major findings. First, having a positive attitude towards nutrition education in the future is influenced by the instructions from teachers in classes during elementary school and middle school. Second, having nutritionally balanced diets in the future is affected by family conversation about foods during elementary school. These results suggest that effective nutrition education policies for children should not only promote in-class nutrition education, but also look for close cooperation with the students’ family members.

## Figures and Tables

**Table 1:** Summary of the survey participants in the sample (*n* = 1535).

	Male		Female	
Age	Frequency	%	Frequency	%
Total	676	44.04	859	55.96
20–29	62	4.04	74	4.82
30–39	90	5.86	111	7.23
40–49	122	7.95	145	9.45
50–59	99	6.45	146	9.51
60–69	131	8.53	155	10.10
70+	172	11.21	228	14.85

**Table 2:** Summary statistics of the key variables (*n* = 1535).

Variable	Mean	SD
Dependent Variables		
Interests in nutrition education	1.891	0.922
Having nutritionally balanced meals	2.028	0.750
Independent Variables		
Elementary: Participation in farming experience	2.463	1.636
Elementary: Instructions by teachers	3.086	1.507
Elementary: Conversation with family on the food during eating	3.190	1.201
Elementary: Family’s effort to cook likable food	2.465	1.219
Middle: Participation in farming experience	3.038	1.644
Middle: Instructions by teachers	3.398	1.464
Middle: Conversation with family about food while eating	3.280	1.204
Middle: Family’s effort to cook likable food	2.678	1.283
High: Participation in farming experience	3.575	1.596
High: Instructions by teachers	3.792	1.406
High: Conversation with family about food while eating	3.453	1.217
High: Family’s effort to cook likable food	2.927	1.341

Note: each question shows the respondents’ agreement with the statement, with minimum value of 1 indicating strong agreement and maximum value of 5 indicating strong disagreement.

**Table 3:** Logistic regression on the effects of nutrition education on attitude towards nutrition education.

Dep. Variable: Attitude towards Nutrition Education	Coefficient (Standard Error)	95% Confidence Interval
Farming experience (elementary)	−0.026 (0.056)	(−0.136–0.084)
Instructions by teachers (elementary)	0.122 * (0.067)	(−0.008–0.253)
Family conversation (elementary)	0.137 (0.088)	(−0.035–0.310)
Family cooking effort (elementary)	0.086 (0.091)	(−0.092–0.265)
Farming experience (middle)	0.090 (0.070)	(−0.048–0.227)
Instructions by teachers (middle)	0.202 ** (0.080)	(0.046–0.358)
Family conversation (middle)	0.034 (0.101)	(−0.165–0.233)
Family cooking effort (middle)	−0.026 (0.107)	(−0.235–0.183)
Farming experience (high)	−0.038 (0.065)	(−0.165–0.089)
Instructions by teachers (high)	−0.099 (0.073)	(−0.241–0.044)
Family conversation (high)	0.113 (0.093)	(−0.069–0.295)
Family cooking effort (high)	0.028 (0.089)	(−0.148–0.203)
Gender	−0.667 *** (0.131)	(−0.924–−0.410)
Age	−0.087 *** (0.023)	(−0.132–−0.042)
Sample Size	1529	
Log likelihood	−751.70	
LR	129.42	

Note: Standard errors are shown in parentheses. * *p* < 0.10, ** *p* < 0.05, *** *p* < 0.01.

**Table 4:** Ordered logistic regression on the effects of nutrition education on having nutritionally balanced meals.

Dep. Variable: Having Nutritionally Balanced Meals	Coefficient (Standard Error)	95% Confidence Interval
Farming experience (elementary)	0.008 (0.045)	(−0.081–0.098)
Instructions by teachers (elementary)	0.056 (0.054)	(−0.049–0.162)
Family conversation (elementary)	0.138 * (0.071)	(−0.002–0.277)
Family cooking effort (elementary)	0.043 (0.073)	(−0.101–0.187)
Farming experience (middle)	0.026 (0.055)	(−0.081–0.133)
Instructions by teachers (middle)	−0.049 (0.062)	(−0.171–0.072)
Family conversation (middle)	0.046 (0.081)	(−0.113–0.205)
Family cooking effort (middle)	0.035 (0.085)	(−0.132–0.203)
Farming experience (high)	0.063 (0.050)	(−0.035–0.161)
Instructions by teachers (high)	−0.011 (0.055)	(−0.118–0.097)
Family conversation (high)	0.022 (0.072)	(−0.120–0.163)
Family cooking effort (high)	−0.002 (0.071)	(−0.143–0.139)
Gender	−0.416 *** (0.104)	(−0.619–−0.212)
Age	−0.182 *** (0.018)	(−0.217–−0.146)
Sample Size	1533	
Log likelihood	−1662.33	
LR	171.44	

Note: Standard errors are shown in parentheses. * *p* < 0.10, *** *p* < 0.01.

## Data Availability

Restrictions apply to the availability of these data. Data were obtained from the Social Science Japan Data Archisve, Center for Social Research and Data Archives, Institute of Social Science, The University of Tokyo and are available at https://csrda.iss.u-tokyo.ac.jp (accessed on 29 October 2021), with the permission of the Social Science Japan Data Archive, Center for Social Research and Data Archives, Institute of Social Science, The University of Tokyo.
